# Molecular Competition in G1 Controls When Cells Simultaneously Commit to Terminally Differentiate and Exit the Cell Cycle

**DOI:** 10.1016/j.celrep.2020.107769

**Published:** 2020-06-16

**Authors:** Michael L. Zhao, Atefeh Rabiee, Kyle M. Kovary, Zahra Bahrami-Nejad, Brooks Taylor, Mary N. Teruel

**Affiliations:** 1 Department of Chemical and Systems Biology, Stanford University, Stanford, CA, USA; 2 Department of Biochemistry and the Drukier Institute for Children’s Health, Weill Cornell Medicine, New York, NY, USA; 3 Lead Contact

## Abstract

Terminal differentiation is essential for the development and maintenance of tissues in all multi-cellular organisms and is associated with permanent exit from the cell cycle. Failure to permanently exit the cell cycle can result in cancer and disease. However, the molecular mechanisms and timing that coordinate differentiation commitment and cell cycle exit are not yet understood. Using live, single-cell imaging of cell cycle progression and differentiation commitment during adipogenesis, we show that a rapid switch mechanism engages exclusively in G1 to trigger differentiation commitment simultaneously with permanent exit from the cell cycle. We identify a molecular competition in G1 between when the differentiation switch is triggered and when the proliferative window closes that allows mitogen and differentiation stimuli to control the balance between terminally differentiating cells produced and progenitor cells kept in reserve, a parameter of critical importance for enabling proper development of tissue domains and organs.

## In Brief

Zhao et al. show that in terminally differentiating cells, a rapid switch mechanism engages exclusively during a G1 period to trigger simultaneous differentiation commitment and permanent cell cycle exit. A molecular competition in each G1 period allows cells to control the balance the terminally differentiating cells produced and progenitor cells kept in reserve.

## Graphical Abstract

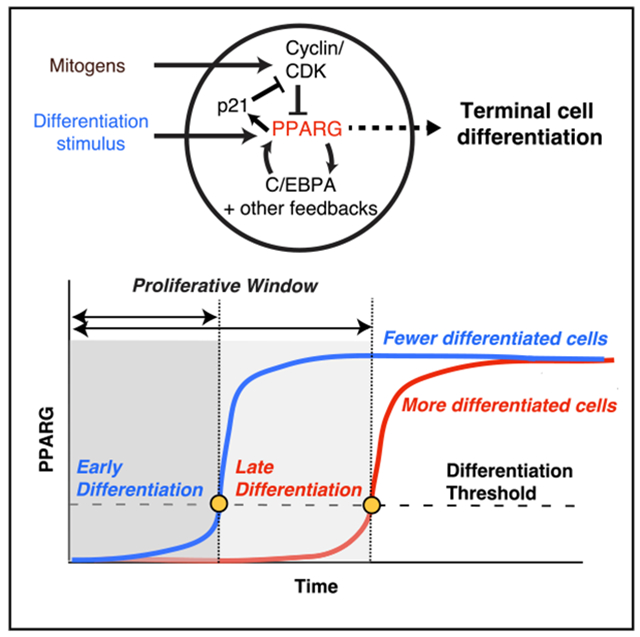

## INTRODUCTION

Terminal differentiation is essential for developing, maintaining, and regenerating tissues in humans and other multi-cellular organisms and is the mechanism by which neurons, skeletal muscle cells, adipocytes (fat cells), and many other critical cell types are generated (Ruijtenberg and van den Heuvel, 2016). Terminal differentiation typically requires that proliferative progenitor cells permanently exit the cell cycle. Failure of terminally differentiated cells to permanently exit the cell cycle can lead to disease and is a hallmark of cancer (Ghaben and Scherer, 2019; Ruijtenberg and van den Heuvel, 2016). However, despite the fundamental importance of coordinating terminal cell differentiation with permanent cell cycle exit, whether, how, and when this occurs has not been clear (Buttitta and Edgar, 2007; Hardwick et al., 2015; Soufi and Dalton, 2016).

A main bottleneck in understanding the relationship between cell cycle exit and terminal differentiation is the great variability in whether and when individual progenitor cells in the same population proliferate or differentiate during the several-day-long differentiation process, making it difficult to answer timing questions using traditional bulk cell approaches. To overcome this challenge, methods are needed that can simultaneously track cell cycle and differentiation progression live in individual cells in order to measure whether and when during the multi-day differentiation time course an individual cell commits to irreversibly differentiate. However, such live-cell imaging studies for terminal cell differentiation have to our knowledge not yet been made.

Here we sought to understand the molecular mechanisms underlying when and how cells coordinate the commitment to terminally differentiate and exit the cell cycle and how such coordination may control the number of terminally differentiated cells produced. We use adipogenesis as a model system for terminal differentiation because the cell cycle is known to regulate adipogenesis, and the validity of using *in vitro* cell models for adipogenesis studies has been corroborated by *in vivo* studies (Ghaben and Scherer, 2019; Jeffery et al., 2015; Tang et al., 2003). We start by validating that a threshold level of fluorescently tagged endogenous PPARG protein can be used in live cells to mark the precise time when preadipocytes irreversibly commit to terminally differentiate. By combining this live-cell PPARG sensor with a reporter to mark the G1 phase (Sakaue-Sawano et al., 2008), we establish a method to simultaneously track both cell cycle progression and the precise commitment point to terminally differentiate. Markedly, we show that cells commit to terminally differentiate exclusively in G1 by triggering a PPARG-driven switch and that this same switch also triggers permanent cell cycle exit by rapidly inducing high expression and increased stability of the CDK inhibitor p21. Thus, cells become post-mitotic precisely when they commit to terminally differentiate. Importantly, we show that after a differentiation stimulus has been added, cells undergo a competition during each G1 period between whether a cell starts the next cell cycle or, alternatively, terminally differentiates and permanently stops future cell divisions. We show that the levels of p21 and cyclin D, as well as the mitogen and adipogenic stimuli that induce expression of these proteins, influence the outcome of this competition by controlling the duration of G1, as well as the rate of PPARG increase, during G1. The existence of this competition in G1 means that the cell is able to control both the number of terminally differentiated cells produced and the number of progenitor cells that are kept in reserve. Thus, G1 competition represents a control principle that can explain how a tissue can regulate the number of terminally differentiated cells produced while maintaining pools of progenitor cells at similar levels.

## RESULTS

### Development of a Live-Cell Readout for the Precise Time When a Cell Commits to Terminally Differentiate

**Figure 1.**
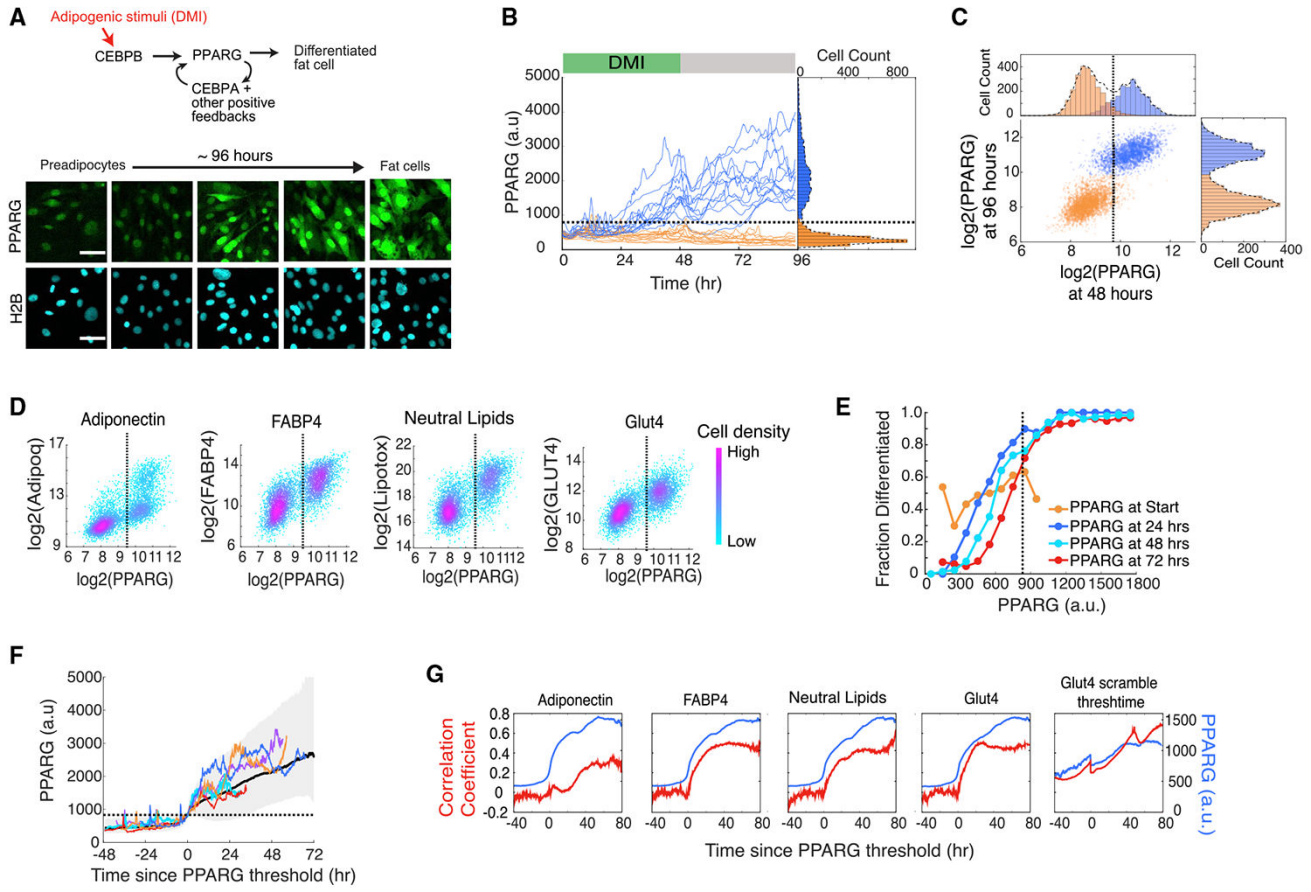
Live-Cell Analysis of Endogenous PPARG Expression Shows that There Is a Precise Time When Cells Irreversibly Commit to Differentiate (A) Cells expressing endogenous citrine-PPARG were differentiated using the standard 96 h DMI protocol. Scale bar, 50 μm. (B) Results of a typical experiment in which thousands of single cells were stimulated to differentiate. In the standard 96 h DMI differentiation protocol, an adipogenic cocktail (DMI) is added to the cell medium for 48 h (green horizontal bar). Then the medium is replaced with fresh medium containing just insulin for another 48 h (grey horizontal bar). Thirty single-cell traces are shown as examples. Representative of four biological replicates. (C) Scatterplot using data from (B) showing PPARG levels for each cell at 48 h, just before the DMI stimulus was removed, and at 96 h. (D) Citrine-PPARG cells were differentiated using the DMI protocol, and immunocytochemistry for adipocyte markers was performed at 96 h (for each scatterplot, n > 4,000 cells, representative of three biological replicates). (E) The time courses from (B) were split into equal-width bins by their PPARG values at 0, 24, 48, and 72 h. The fraction of differentiated cells represents the number of cells that crossed the PPARG threshold at the end of the experiment divided by the number of cells in the bin. (F) Differentiating cells from (B) were computationally aligned so that the zero time point represents the time when the cell crossed the PPARG threshold. Plot shows five representative single-cell traces, the median (solid black line), and the 5th to 95th percentiles (shaded region). (G) PPARG time courses from the cells that differentiated after 96 h in (D) were computationally aligned as in (F) and plotted (blue curves). At each aligned time point, the Pearson correlation coefficient between the aligned PPARG values and the endpoint immunofluorescence values for adipocyte markers was calculated (red curves). As a comparison, PPARG values for the Glut4 panel were aligned to a randomized PPARG threshold crossing point. The randomized crossing point was generated by scrambling the vector of measured threshold points for each cell so that each threshold point is matched with different cell. In (B)–(F), the dotted line represents the calculated PPARG threshold for that experiment. Cells with PPARG levels above and below the threshold level are defined to be differentiated (PPARG High) and undifferentiated (PPARG Low), respectively. See also Figure S1.

A major limiting factor in understanding the relationship between the cell cycle and terminal differentiation has been the lack of a quantitative live-cell readout that can mark the precise time point when a cell commits to terminally differentiate (Buttitta and Edgar, 2007). We thus started by establishing such a live-cell readout. During adipogenesis, expression of PPARG, the master transcriptional regulator of adipogenesis, is driven by both external input signals and internal positive feedback loops (Ahrends et al., 2014; Rosen and Spiegelman, 2014) (Figure 1A, top). Previously, we had used CRISPR-mediated genome editing to tag endogenous PPARG with citrine(YFP) fluorescent protein in an OP9 preadipocyte cell line (Figure 1A, bottom) (Bahrami-Nejad et al., 2018). We now validate that a threshold in PPARG levels can be used as a live-cell readout for the precise time when a cell commits to terminally differentiate.

To determine whether there is a threshold for differentiation, it is critical to remove the differentiation stimulus at an intermediate time point and test whether a cell can continue on to reach and maintain a distinct differentiated state days later (Bahrami-Nejad et al., 2018). We induced differentiation by applying the standard DMI adipogenic hormone cocktail that mimics glucocorticoids and GPCR signals that raise cAMP to preadipocytes for 48 h (see STAR Methods). The distribution of PPARG levels at the end of the 96 h differentiation protocol is bimodal, with high and low peaks representing the differentiated and undifferentiated cells, respectively (Figures 1B and 1C). Comparing each cell’s PPARG level at 96 h with its PPARG level at 48 h before the stimulus was removed showed that the level of PPARG before stimulus removal could indeed predict, with a less than 5% false positive rate, whether a cell would go on to differentiate 2 days later, confirming that a threshold level in PPARG can predetermine the cell’s final fate (Figures 1C and S1A-S1C). This threshold level is calculated at the end of each experiment as the center between the two peaks in the PPARG histogram at the 48 h time point (Figures 1B and 1C, black dashed line; see STAR Methods).

We validated that cells with PPARG levels above the threshold are in the differentiated adipocyte state by using different well-established markers of mature adipocytes (Figure 1D). Furthermore, we showed that PPARG levels are predictive of final fate independently of when cells pass the threshold for terminal differentiation and that cells with higher PPARG levels maintain a higher probability to differentiate at different time points throughout adipogenesis (Figure 1E). Interestingly, the probability to differentiate could not be predicted by PPARG levels at the start of the experiment (Figure 1E), suggesting that terminal differentiation fate is determined mostly by differences in signaling strength rather than initial PPARG expression.

Previous work showed that a positive feedback-driven bistable switch mechanism between PPARG and several co-regulators can amplify PPARG expression (Ahrends et al., 2014; Wu et al., 1999) (Figure 1A, top). To determine whether such a positive feedback-driven bistable switch is responsible for cells to reach the PPARG threshold, we computationally aligned single-cell traces to the time when each cell crosses the PPARG threshold. Markedly, the aligned time courses show a sharp sigmoidal increase from a slow rate of PPARG increase before the PPARG threshold to a fast rate after that time point (Figures 1F and S1D). This observed switch from low to high PPARG levels exactly at the time point at which the PPARG threshold is reached argues that the PPARG threshold marks the precise time point when the bistable PPARG switch mechanism is triggered.

Early in adipogenesis, before cells reach the threshold, PPARG levels are not correlated with endpoint measurements of adipocyte markers (Figure 1G). However, once the threshold is reached, PPARG levels sharply switch to being positively correlated, supporting that crossing the PPARG threshold marks a short time window of PPARG self-amplification that causes an irreversible commitment to the future terminally differentiated adipocyte state (see also Figures S1D and S1E). Taken together, these different experiments validate that a threshold level can be used to mark a precise timepoint when progenitor cells commit to terminally differentiate, even before the markers of mature fat cells can be measured. When adipogenic stimuli are removed, cells that pass the PPARG threshold go on to terminally differentiate, while cells below the threshold return to the undifferentiated progenitor state (Bahrami-Nejad et al., 2018). Thus, fluorescently tagged endogenous PPARG can be used to directly address the questions of when a cell commits to terminally differentiate and what the connection is between the commitment to terminally differentiate and permanent exit from the cell cycle.

### Simultaneous Single-Cell Analysis Shows that Further Entry into the Cell Cycle Is Blocked Once a Cell Reaches the Differentiation Commitment Point in G1

**Figure 2.**
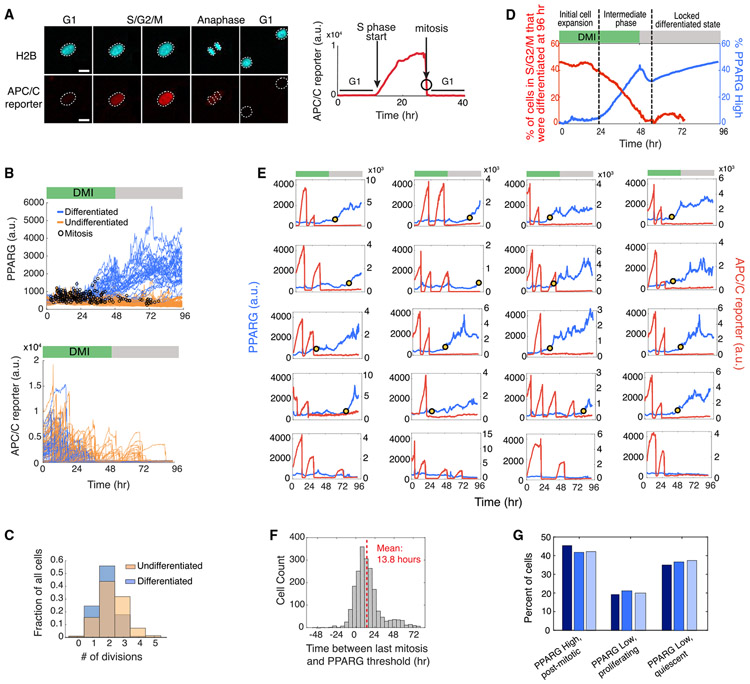
Cells Commit to Terminally Differentiate Exclusively during G1 Phase (A) Dual-reporter cells stably expressing APC/C-reporter-mCherry(RFP) and citrine(YFP)-PPARG. Anaphase is shown both by a split in the H2B-mTurquoise signal (top images) and by a sharp drop in APC/C reporter signal (bottom time course). White outlines mark the position of nuclei in cells after anaphase. Scale bar, 20 μm. (B) Cells were induced to differentiate using the standard 96 h DMI protocol. The dual-reporter cells allow simultaneous measurement in thousands of single cells of differentiation state using PPARG levels (left) and cell cycle state using the APC/C sensor (right). The time points at which mitosis occurred were determined by using the split in H2B signal (black open circles). Representative of four biological replicates. (C) Comparison of the number of observed mitotic events that occurred in cells that were differentiated versus cells that remained undifferentiated at the end of the 96 h experiment shown in (B). (D) Plot showing how the fraction of cells in S/G2/M (red) or with PPARG levels higher than the threshold (blue) varies during a 96 h differentiation time course induced by DMI. (E) Examples of PPARG (blue) and APC/C reporter (red) time courses obtained in the same single cell. Cells were stimulated to differentiate using the standard 96 h DMI protocol. The yellow dot in each plot marks the time at which that cell reached the PPARG threshold and irreversibly committed to the differentiated state. Bottom row shows examples of three undifferentiated/proliferating cells and one undifferentiated/quiescent cell that no longer proliferates even after a serum refresh at 48 h. (F) Histogram of the difference between the time when the PPARG threshold is crossed and when mitosis last occurred for each cell in the experiment shown in (B). The PPARG threshold is reached on average ~14 h after the last mitosis is completed. The median value is ~11 h. Negative values indicate cells that reached the PPARG threshold before the last mitosis was completed. (G) Percentage of differentiated/post-mitotic, undifferentiated/proliferating, and undifferentiated/quiescent cells generated in three independent DMI-induced differentiation experiments. Cells with PPARG levels above and below the threshold level are defined to be differentiated (PPARG High) and undifferentiated (PPARG Low), respectively, as described in Figure 1. See also Figures S2-S4.

We next made a dual-reporter cell line by transfecting a FUCCI cell cycle reporter (Sakaue-Sawano et al., 2008) into the citrine-PPARG preadipocyte cell line described in Figure 1A (Figure 2A; Videos S1 and S2). The fluorescent mCherry(RFP) signal of the cell cycle reporter (hereafter referred to as the APC/C reporter) rapidly drops in mitosis and starts to increase only close to the end of G1 phase, when the second APC/C^CDH1^, which is active during G1, is rapidly inactivated (Cappell et al., 2016). To determine when terminal cell differentiation occurs relative to the cell cycle, we tracked PPARG expression and APC/C reporter time courses over 4 days of differentiation. The resulting cell trajectories show that cells that go on to terminally differentiate have fewer cell cycles and exit the last mitosis earlier (Figures 2B and 2C) compared with cells that do not end up differentiating. Such an inverse relationship between proliferation and terminal differentiation can be represented in a cumulative plot comparing the percentage of cells still in S/G2/M versus the percentage of cells that have crossed the PPARG threshold for terminal differentiation, as a function of time after DMI stimulation (Figure 2D). Control experiments that characterized the cell plating conditions used (Figures S3A and S3B) support that the lower differentiation we observed in cycling cells is not due to PPARG levels simply being diluted more in cells that cycle more often (Figures S3C and S3D).

Strikingly, when visually inspecting thousands of single-cell traces in preadipocytes induced to differentiate, we observed no new cell cycle entry after the PPARG level in a cell increased above the threshold for terminal differentiation (Figure 2E, yellow dot), arguing that permanent cell cycle exit is forced on cells when they reach the commitment point to terminally differentiate. We observed that in many cells, PPARG levels increase already during S/G2/M phase, but cells reach the PPARG threshold for terminal differentiation almost exclusively in G1 phase (see also Figures S3E and S3F). A histogram analysis shows that a cell needs approximately 14 h in G1 phase after the last mitosis to commit to the terminally differentiated state (Figure 2F). It should be noted that our live dual-reporter method, with which we can measure cell cycle and differentiation progression simultaneously, allows us to distinguish between cells that become (1) post-mitotic and differentiated, (2) undifferentiated and proliferating, or (3) undifferentiated and quiescent (Figure 2G). As an example of a quiescent cell, the bottom right plot in Figure 2E shows a cell that remains undifferentiated but ceases to proliferate even when serum is refreshed at 48 h.

An interesting result from this analysis is that preadipocytes undergo a variable number of cell divisions before they differentiate (Figures 2C and 2E), arguing that terminal differentiation of adipocytes does not occur after a fixed number of cell divisions before differentiation, as has been previously suggested (Tang et al., 2003). As the previous study relied on averaged, population-based measurements, the variable number of mitoses in different cells could likely not be resolved without live single-cell analysis. Not only does the number of cell cycles vary, but there is also great variability in the time after stimulation when cells start to increase PPARG levels, and also in the time cells spend in G1 before cells reach the PPARG threshold for terminal differentiation (see also Figure S4). Taken together, our results show that terminal adipocyte differentiation occurs after a variable rather than fixed number of cell cycles and that cells commit to differentiate almost exclusively during G1 phase. Furthermore, the time cells need in G1 to reach the differentiation commitment point is variable, and during S/G2/M, PPARG increases are suppressed. Strikingly, our dual-reporter timecourses show that cells permanently exit the cell cycle at precisely the same time when they pass the commitment point for terminal differentiation.

### PPARG Regulates Terminal Cell Cycle Exit by Inducing p21 and FKBPL

**Figure 3.**
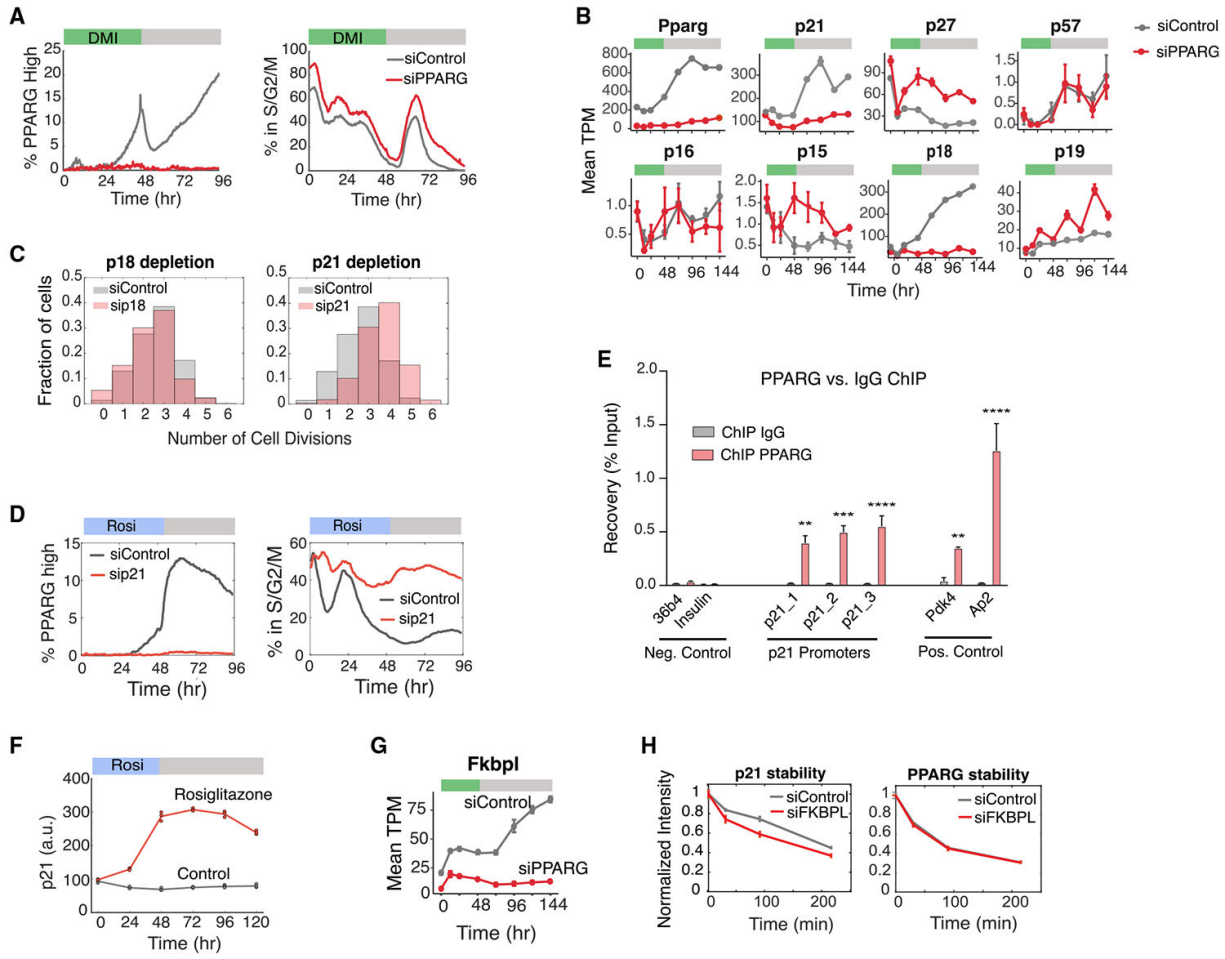
PPARG Directly Upregulates Expression of the CDK Inhibitor p21 (A) Cells transfected with PPARG or control siRNA were stimulated to differentiate by the standard 96 h DMI protocol. The percentage of cells in S/G2/M phases at each time point is calculated by counting the cells that expressed the APC/C reporter during the 96 h differentiation period divided by the total number of cells. The percentage of PPARG high cells was assessed by counting the cells with PPARG levels above the threshold divided by the total number of cells at the respective time point. Approximately 5,000 cells were analyzed per experiment. Representative of three biological replicates. (B) Wild-type OP9 cells transfected with PPARG or nontargeting siRNA were stimulated to differentiate with DMI. RNA samples were collected every 24 h for 144 h. Bar plots show mean ± 1 SD for three technical replicates. (C) Dual-reporter cells transfected with p21, p18, or nontargeting siRNAs were stimulated to differentiate with DMI. The number of cell divisions per cell is reported in the normalized histograms. Representative of two biological replicates. (D and E) Wild-type OP9 cells were stimulated to differentiate by addition of 1 μM rosiglitazone for 48 h. (D) p21 levels at different time points were measured by immunocytochemistry. Approximately 5,000 cells were analyzed per experiment. The values of three technical replicates (points) are plotted on top of the mean (line). (E) Chromatin immunoprecipitation (ChIP) of PPARG was performed, followed by qPCR. Three sites on the p21 promoter are shown. The promoters of insulin and Arbp/36b4 served as negative controls, and known PPARG target genes Fabp4/aP2 and Pdk4 were used as positive controls. Data are normalized to a nontargeting genomic site and IgG enrichment. Two-way ANOVA with Bonferroni’s multiple comparisons test was applied for statistical analysis. Values show mean ± SEM and are representative of two biological replicates. p < 0.05, **p < 0.01, ***p < 0.001, and ****p < 0.0001. (F) Wild-type OP9 cells were transfected with p21 or control siRNA, stimulated to differentiate by addition of 1 μM rosiglitazone, and analyzed as in (A). (G) FKBPL expression under nontargeting versus PPARG knockdown were obtained from the RNA-seq data in (B). Data are reported as TPM, mean ± 1 SD. (H) Wild-type OP9 cells were transfected with FKBPL or nontargeting siRNAs and stimulated to differentiate with DMI. Stability of p21 and PPARG were assessed by adding 30 μM cycloheximide to the media 24 h after DMI addition and then fixing and staining for protein levels at different subsequent times. Approximately 5,000 cells were analyzed per experiment. Data are plotted as mean ± 1 SD of three technical replicates.

The dual-reporter time courses in Figures 2B-2E showed that DMI-stimulated cells that stopped proliferating earlier also had consistently higher levels of PPARG, supporting that PPARG may suppress the cell cycle and also regulate permanent cell cycle exit. To test for this, we carried out small interfering RNA (siRNA) experiments which showed that depletion of PPARG indeed results in an increase in the percentage of proliferating cells at all time points throughout the differentiation process (Figure 3A). On the basis of our observation that differentiation commitment occurs almost exclusively in G1 phase (Figures 2E and 2F) and out of a state with low CDK2 activity (Figures S3E and S3F), we hypothesized that PPARG may increase the expression of one of the CDK inhibitors, which may then slow or inhibit entry into the next cell cycle.

We sought to identify putative inhibitors of proliferation by performing comparative RNA sequencing (RNA-seq) analysis using cells transfected with siRNA targeting PPARG or control siRNA and collected at different time points during a 144 h DMI differentiation protocol. When we examined mRNA expression profiles of canonical CDK inhibitors, we identified two that were strongly regulated by PPARG expression, p18 and p21 (Figure 3B). To determine whether p18 and p21 mediate cell-cycle arrest during adipogenesis, we carried out siRNA experiments and found that p21, but not p18, knockdown leads to an increase in proliferation (Figure 3C). Furthermore, p21 is required for PPARG both to mediate terminal cell differentiation and to suppress proliferation (Figure 3D). We tested whether PPARG could regulate p21 expression directly by performing chromatin immunoprecipitation sequencing (ChIP-seq) experiments, which revealed significant binding of PPARG to the promoter of p21 during adipogenesis induced by DMI stimulation (Figure 3E). To further test whether the effect of PPARG on p21 is direct, we added rosiglitazone, a small molecule that directly activates PPARG, which led to a robust increase in p21 expression (Figure 3F).

In the same RNA-seq data, we found that PPARG increases the expression of FKBPL (WiSP39), a protein that was shown to stabilize p21 (Jascur et al., 2005) (Figure 3G). To test if p21 could be stabilized by FKBPL during the early stages of adipogenesis, we carried out cycloheximide protein degradation experiments in cells transfected with siRNA targeting FKBPL. Our results showed that knockdown of FKBPL causes a small decrease in p21 half-life but does not affect the half-life of PPARG, supporting that FKBPL does regulate p21 stability during terminal cell differentiation (Figure 3G). Taken together, our results demonstrate that PPARG slows, or stops, the cell cycle during G1 phase by two mechanisms: increasing p21 levels via increasing p21 transcription and by FKBPL-mediated slowing of p21 degradation.

### Commitment to Terminally Differentiate Triggers Immediate p21-Driven Cell Cycle Exit

**Figure 4.**
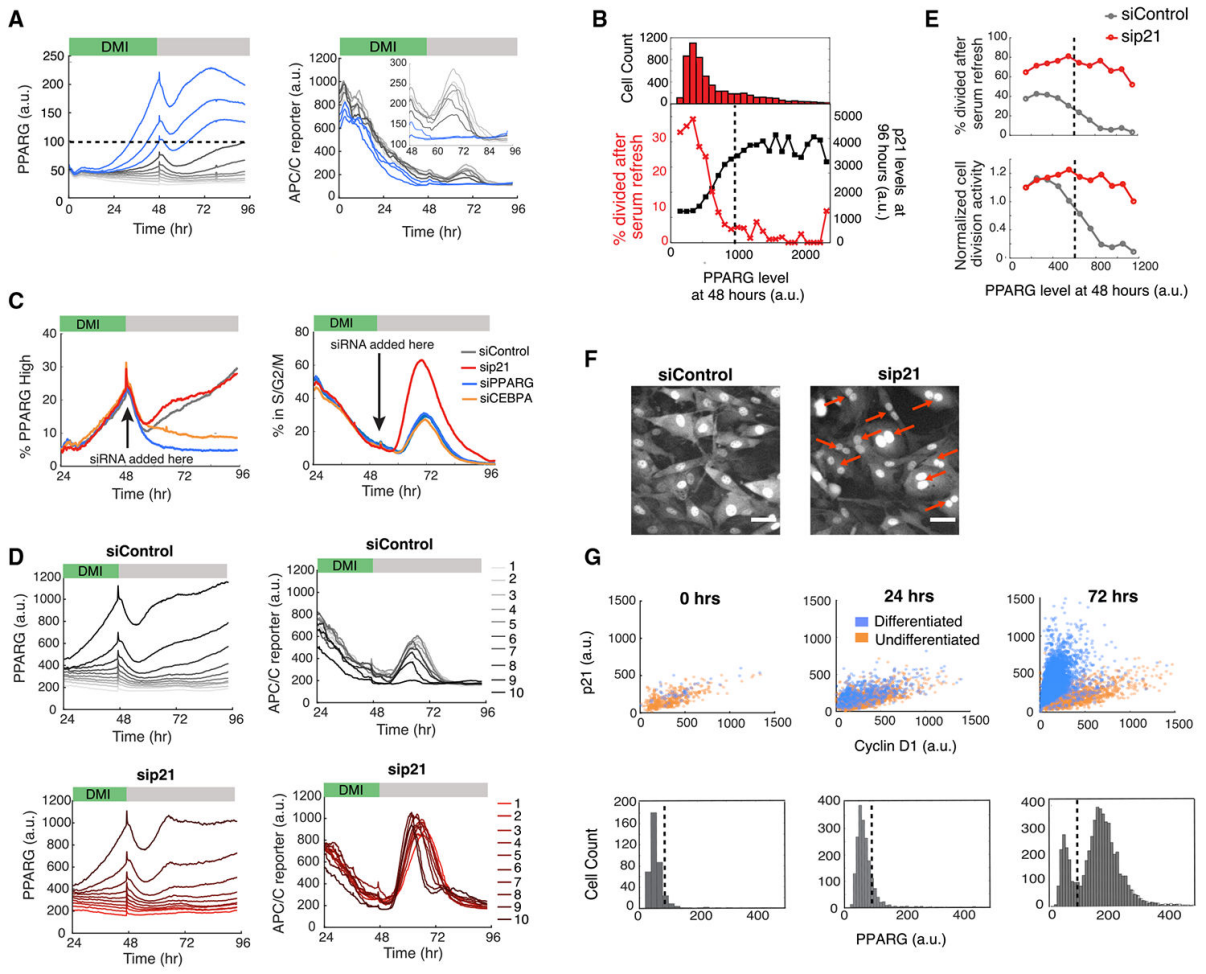
Cells Simultaneously Commit to Differentiate and Become Locked in a Post-mitotic State by PPARG-Induced Maintenance of High Levels of p21 Expression (A) Dual-reporter cells were induced to differentiate using the standard 96 h DMI protocol. Cells were separated into ten bins on the basis of their PPARG levels at 48 h. Plotted lines show mean values for each bin. Inset shows the APC/C reporter signal between 48 and 96 h. Representative of two biological replicates. (B) Dual-reporter cells were induced to differentiate using the standard 96 h DMI protocol and separated into bins according to their PPARG value before serum refresh at 48 h. Cells were fixed and stained for p21 levels at the end of the differentiation time course at 96 h. Bins are from 100 to 2,500 a.u. in 100 a.u. increments. The histogram shows the number of cells in each bin. The plotted points show (red) the fraction of cells that had a minimum of one division in response to the serum refresh at 48 h and (black) the average final p21 level. Representative of two biological replicates. (C) PPARG, CEBPA, p21, and nontargeting siRNA were transfected into the dual-reporter cells 48 h after DMI addition. siRNA knockdown efficiency is shown in Figure S5. Representative of three biological replicates. (D) A similar analysis as described in (A) was performed on the nontargeting and p21-knockdown conditions from (C). (E) Top: a similar analysis as in (B) was performed on the nontargeting and p21 knockout conditions from (D). Bottom: the same data normalized to the first PPARG bin. (F) Images of control and p21-knockdown cells from (C) obtained 48 h after siRNA transfection (96 h). Red arrows indicate representative multi-nucleated cells. Scale bar, 50 μm. (G) OP9 cells were induced to differentiate with rosiglitazone. Cyclin D1, p21 and PPARG levels were assessed by immunocytochemistry. Representative of two biological replicates. In (A), (B), (E), and (G), the dotted black line shows the PPARG threshold calculated for that experiment. See also Figure S5.

We next focused on the question of how preadipocyte cells trigger permanent exit from the cell cycle once they pass the PPARG threshold for terminal differentiation. We took advantage of the variable increases in PPARG levels between cells in the population following DMI stimulation and grouped cells into ten bins according to their expression level of PPARG at 48 h, right before the DMI-containing medium is replaced with fresh medium without DMI (serum refresh) (Figure 4A). We found that the cells whose PPARG levels stayed below the threshold (gray lines) proliferated in response to the serum refresh at 48 h, as measured by an increase in APC/C reporter signal. However, cells that had passed the PPARG threshold (blue lines) showed no significant APC/C reporter response, demonstrating that cells that have crossed the threshold for terminal differentiation lose their ability to proliferate in response to the serum refresh. We further confirmed this result by calculating the fraction of cells that divided in response to the serum refresh (Figure 4B, red). Together, these results argue that crossing the PPARG threshold marks the time when cells permanently enter a post-mitotic state.

We next investigated how p21 levels in individual cells change relative to PPARG levels. We differentiated dual-reporter cells by using the standard 96 h DMI protocol and fixed and stained the cells at the end of the experiment for p21 expression (Figure 4B, black). We binned the time courses by their PPARG level at 48 h, which corresponds to approximately the time when cells decide to differentiate, and found that cells that had crossed the PPARG threshold at 48 h had high final p21 levels, arguing that p21 is high in cells that terminally differentiate and is low in cells that fail to differentiate. This result suggests that an increase in p21 occurs in all differentiating cells and is responsible for the observed exit to quiescence of differentiating cells.

To directly test for a role of p21 in suppressing proliferation after the differentiation threshold has been reached, we used siRNA to acutely knock down p21 late in adipogenesis at the 48 h time point. As a control, we also knocked down PPARG and CEBPA, a required co-activator of PPARG expression that is needed for cells to reach the threshold for differentiation (Bahrami-Nejad et al., 2018; Wu et al., 1999). Among the three regulators, only knockdown of p21 resulted in a significant increase in cell cycle activity in the already differentiated cells (Figure 4C). To quantitatively analyze this result, we binned cells by their PPARG levels at 48 h (Figure 4D). When control siRNA is transfected in at 48 h after the differentiation commitment point had been reached in most cells, APC/C reporter signals remain suppressed after serum refresh, even in cells with PPARG levels above the threshold (siControl, bin 10; Figure 4D, top panels), consistent with a lack of proliferation. However, in striking contrast, acute knockdown of p21 expression at 48 h (sip21, Figure 4D, bottom panels) results in the differentiated cells being unable to maintain the post-mitotic state. Plotting the percentage of cells in the cell cycle versus the level of PPARG further illustrates that if p21 is knocked down, cell divisions cannot be blocked even in cells past the differentiation threshold with high PPARG levels (Figure 4E). Thus, high p21 is required for differentiated cells to maintain the post-mitotic state. Furthermore, our findings that PPARG directly increases p21 levels (Figure 3) and that high PPARG levels become self-sustaining after commitment (Figure 1) explain how p21, which has a short protein and mRNA half-lives of less than 30 min (Yang et al., 2017), can be continuously maintained at a high level in order to keep differentiated adipocytes permanently in a post-mitotic state. Notably, when we examined images of cells in which p21 had been depleted late in adipogenesis after the cells had crossed the PPARG threshold, we found that the cells were enriched for multinucleation events (Figure 4F). This result suggests that a critical role of p21 is to permanently prevent cell division after cells have terminally differentiated in order to prevent mitotic defects.

Finally, it was recently shown that the ratio of p21 versus cyclin D1 in the nucleus can predict retinoblastoma (Rb) hyperphosphorylation and re-entry into the cell cycle (Yang et al., 2017). We determined whether the PPARG-induced increase in p21 expression shifts the p21/cyclin D1 ratio toward high p21 in order to keep Rb dephosphorylated and ensure that cells do not enter the cell cycle when mitogen stimuli increase cyclin D1 levels. We found that the ratio of p21 to cyclin D1 in individual cells does become strongly skewed toward p21 when PPARG levels go above the threshold during adipogenesis (Figure 4G), providing an explanation of how differentiated cells can maintain a robust arrested state. We conclude that a PPARG-driven rapid differentiation switch is exclusively triggered in G1 to commit preadipocytes to differentiate and that the same differentiation switch simultaneously triggers permanent cell cycle exit with PPARG first inducing and then maintaining p21 expression.

### An Ongoing Competition during G1 between Terminal Differentiation and Continued Proliferation

**Figure 5.**
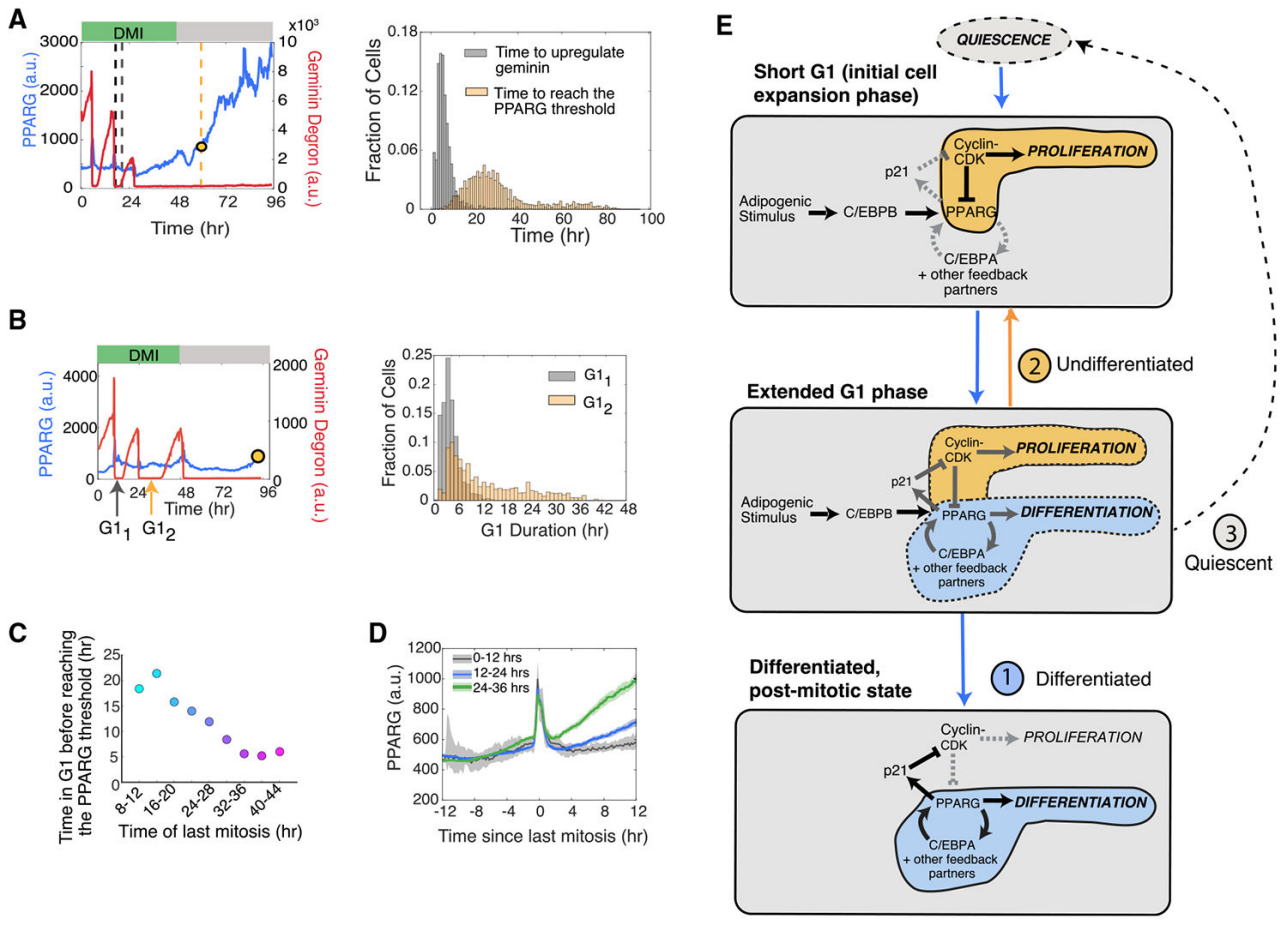
Adipogenic Stimuli Initiate a Competition between the Commitment to Differentiate and Entry into the Next Cell Cycle during a Gradually Extending G1 Phase (A) Comparison of the time to commit to the next cell cycle versus time to commit to differentiation in cells that underwent two or three mitoses before differentiating. Right: the end of the second to last mitosis was used as the starting reference time for each cell. Left: histograms comparing the two times measured in the same cell (data from Figure 2B; n > 4,000 cells, representative of four biological replicates). (B) Left: schematic showing which G1 periods were compared. Right: histograms of the durations of the first and second G1 periods in cells from (A) that have undergone at least three divisions. See also Figure S6. (C) Differentiated cells from Figure 2B were categorized into nine bins on the basis of the time the cell underwent its last mitosis. Plot showing the average time of the last mitosis versus the average time it took for cells in that bin to increase PPARG levels to the differentiation commitment point. (D) Differentiated cells from Figure 2B were separated into three groups on the basis of when they last exited mitosis after differentiation stimulus was applied. The traces were aligned by the last mitosis frame. The median PPARG levels were plotted for each group (dark line). Shaded region represents the 95th confidence interval. Note that the spike in the PPARG levels at aligned time point 0 is due to aligning the time courses to mitosis. At this time point, there is an undefined nuclear PPARG signal because the nuclear envelope is broken down and chromatin (H2B) is condensed. (E) Schematic of the three stages of G1 lengthening in response to adipogenic (DMI) stimuli. See also Figure S6.

The dual-reporter time courses in Figure 2E suggests that there is a competition that takes place during each G1 phase with two outcomes: (1) PPARG levels in a cell reach the threshold for differentiation, and proliferation is suppressed, or (2) the cell enters the next cell cycle, which once again suppresses increases in PPARG. In addition to this competition, there is also a subset of cells that remain or go quiescent without participating in the competition (Figures 2E and 2G). In the cells that compete, G1 phase typically lasts 4 h in proliferating OP9 cells, but differentiating cells typically need at least 14 h in G1 to reach the PPARG threshold and terminally differentiate (Figures 2F and 5A), which suggests that cells need to lengthen G1 in order to have a chance to terminally differentiate. To test for G1 lengthening, we analyzed dual-reporter time courses and found that the second G1 length is indeed typically significantly longer (Figure 5B, right; Figures S6A and S6B). Thus, we conclude that adipogenic stimuli do lengthen G1 and that the lengthening occurs progressively over multiple cell cycles before cells terminally differentiate out of the last G1 phase.

As adipogenic stimuli are known to increase the expression of PPARG and other key adipogenic transcription factors (Rosen and Spiegelman, 2014), we next tested how this might affect the competition in G1. Indeed, the longer cells are exposed to the adipogenic stimuli, as measured by when they had their last mitosis relative to inducing differentiation, the faster the cell can reach the PPARG threshold in G1 (Figure 5C). Figure 5D also shows that the longer a cell is exposed to the differentiation stimulus, the faster it increases PPARG levels. If a cell had its last mitosis between 0 and 12 h after DMI addition, PPARG did not noticeably increase in the subsequent G1. However, a small increase in PPARG can be observed if a cell had its last mitosis 12–24 h after DMI addition, and an even faster increase in PPARG can be observed if a cell had its last mitosis between 24 and 36 h after DMI addition (Figure 5D). Thus, we conclude that PPARG increases in G1 phase are initially very slow but become steeper after 1–2 days of DMI stimulation, which allows the threshold for differentiation to be reached more quickly during G1.

Taken together, our data show that terminal differentiation of adipocytes occurs through a competition in G1 (Figure 5E): When adipogenic stimuli are first applied, preadipocytes initially proliferate with short G1 periods. Adipogenic stimuli then gradually over time extend G1 length in sequential cell cycles and also gradually accelerate, during each G1, the rate at which PPARG increases to the threshold. These two actions help set up a competition after each mitosis between whether cells first reach the threshold for differentiation or first enter the next cell cycle.

### p21 and Cyclin D1 Compete to Regulate the Time When Cells Commit to Differentiate and Thereby Control the Number of Terminally Differentiated Cells Produced

**Figure 6.**
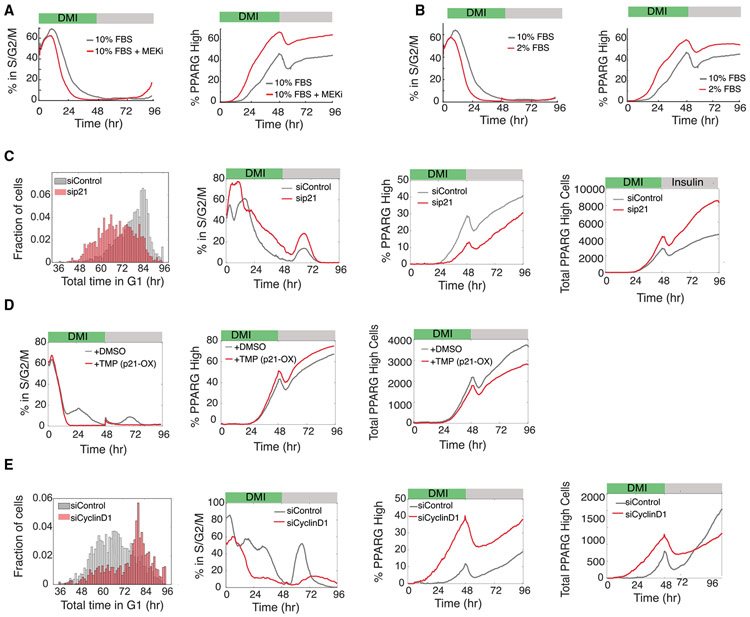
Regulation of the Relative Expression of p21 and Cyclin D1 Delays or Accelerates Differentiation Commitment to Control the Total Number of Differentiated Cells Produced Dual-reporter cells were differentiated using the standard 96-hour DMI protocol: (A) in the presence of a MEK inhibitor (PD0325091) (representative of two biological replicates; (B) in normal (10%) and reduced (2%) serum concentrations (representative of two biological replicates); (C) when transfected with p21 or nontargeting (control) siRNAs (representative of three biological replicates); (D) when stably expressing a DHFR-p21-mCherry fusion protein and in the presence of 10 μM TMP (to increase expression of p21) or DMSO (control) (representative of two biological replicates); and (E) when transfected with cyclin D1 or nontargeting (control) siRNAs (representative of three biological replicates). Time course data were analyzed as in Figure 3A. Histograms show the total time spent in G1 phase for each cell trace across all cell cycles for the respective experimental condition. See also Figure S7.

Up to this point, we had focused on understanding how adipogenic stimuli regulate cell cycle exit and terminal differentiation. We next focus on the role of mitogen stimuli. We started by manipulating the Ras/MEK/ERK signaling pathway, which is activated by most mitogen stimuli. Indeed, when we added a MEK inhibitor to cells stimulated cells with DMI (Figure 6A), we observed as expected that a smaller percentage of cells proliferated and that the time window after stimulation during which cells kept proliferating was shorter. At the same time, the percentage of cells that terminally differentiated was increased, and cells committed to terminally differentiate earlier. Similarly, decreasing the serum concentration along with DMI stimulation reduced the percentage of cells that proliferated and shortened the time window after stimulation in which cells could proliferate (Figure 6B). This was accompanied by an increase in the percentage of terminally differentiated cells, and cells committed to terminally differentiate earlier.

At first glance, the results in Figures 6A and 6B support the generally accepted hypothesis that proliferation and terminal differentiation are opposing processes (Ruijtenberg and van den Heuvel, 2016), and one might therefore expect that mice lacking p21 or p27 should have more proliferating progenitor cells and fewer adipocytes. However, one of the most striking findings from gene knockout studies is that fat tissues of mice with deleted CDK inhibitors p21 and p27 show a synergistic 6-fold increase in the number of adipocytes with smaller effects in single-knockout mice (Naaz et al., 2004). We used live single-cell analysis approaches to understand this conundrum that there are more adipocytes in mice lacking p21 or p27, even though a loss of p21 or p27 is expected to cause increased mitogen-induced proliferation and thus reduced differentiation.

We first confirmed that cells with knocked down p21 *in vitro* spend overall less time in G1 phase and have increased proliferation, consistent with p21 functioning as an inhibitor of proliferation that lengthens G1 (Figure 6C, left). Similar to the effect we had observed with more mitogen stimulation (Figures 6A and 6B), cells with knocked down p21 also show a delayed commitment to terminally differentiate and can thus have more divisions before differentiation (Figure 6C, middle two plots). Furthermore, the p21-mediated increase in the percentage of proliferating cells results in a corresponding decrease in the percentage of differentiated cells (Figure 6C, middle two plots), which can again be explained by short G1 periods providing less opportunity for PPARG levels to increase during each G1. Strikingly, despite the lower percentage of terminally differentiated cells in p21 knockdown conditions, the total number of terminally differentiated cells significantly increased (Figure 6C, right two plots).

We observed the same seemingly paradoxical positive correlation between proliferation and differentiation when we carried out p21 overexpression experiments using a DHFR induction system: the percentage of proliferating cells decreased and the percentage of differentiated cells increased, but there was a lower total number of differentiated cells (Figure 6D). We realized that the positive correlation between the supposedly opposing processes of proliferation and differentiation could be explained by the fact that there is a time window after the differentiation stimulus during which progenitor cells can still proliferate while PPARG levels are increasing but still below the threshold value. However, once cells reach the PPARG threshold and commit to differentiate, they also shut off the cell cycle (Figure 2E). Thus, the increase in the total number of terminally differentiated cells in the p21-knockdown condition can be explained by each progenitor cell with lower p21 undergoing more cell divisions, which suppresses PPARG increases and therefore causes a delay when cells reach the threshold to differentiate. Thus, the puzzling finding of significantly increased adipogenesis and fat mass in mice lacking p21 or p27 can be explained by an increase in the number of cells produced per progenitor cell during a longer window of proliferation in cells lacking CDK inhibitors.

As p21 can affect both G1 and G2 phases, we carried out experiments to test when PPARG-mediated p21 increases are working. We found that the adipogenic DMI stimuli primarily lengthens G1, rather than S/G2/M (Figure S7A), arguing that PPARG-mediated p21 expression acts primarily by lengthening G1 rather than G2. Furthermore, we selectively lengthened S/G2/M by knocking down CDC25B or CDC25C and did not observe a noticeable effect on differentiation outcome (Figures S7B and S7C) (Karlsson et al., 1999).

We found that cyclin D1 knockdown leads to an increase in G1 duration (Figure 6E, left) . Consistent with our model that an increase in G1 duration allows more cells to build up PPARG levels and differentiate, we observed that the cyclin D1 knockdown reduces the percentage of cells that proliferated, increases the percentage of terminally differentiated cells, and shortens the time it takes for cells to terminally differentiate (Figure 6E, middle two plots). There are also fewer terminally differentiated cells ultimately produced, even though the percentage produced is higher (Figure 6E, two right plots). Thus, cyclin D1 and p21 have opposing effects in regulating cell cycle exit and terminal cell differentiation in G1 phase.

### Dual Control by the Strength of Mitogen and Differentiation Stimuli Can Produce More or Fewer Terminally Differentiated Cells while Maintaining Similar Pools of Progenitor Cells

**Figure 7.**
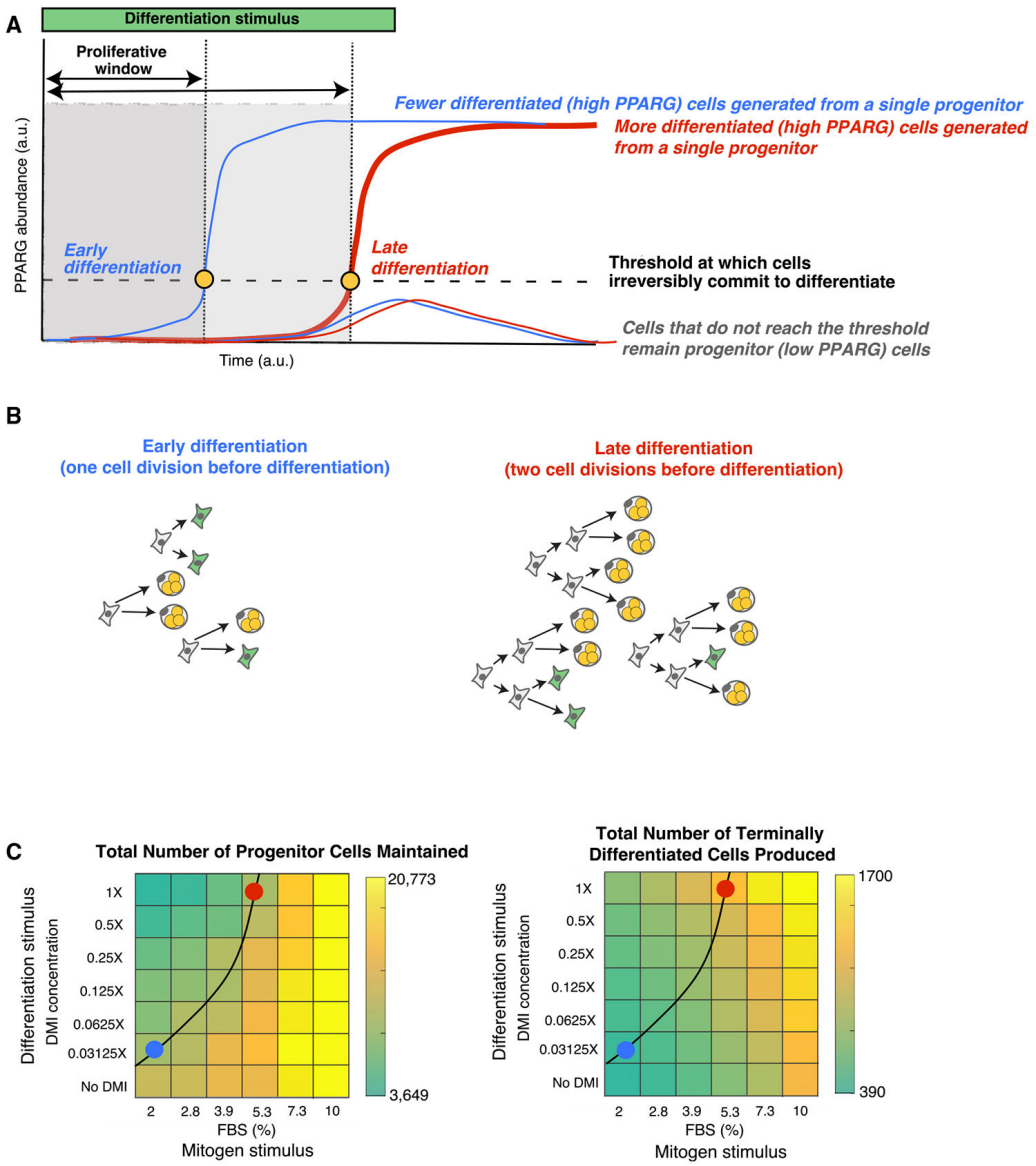
Mitogen and Adipogenic Stimuli Must Be Simultaneously Regulated in Order to Control the Total Number of Differentiated Cells Produced while Keeping the Number of Progenitor Cells at Similar Levels (A) Schematic of how delaying the time to reach the differentiation commitment threshold increases the proliferative window, or time during which progenitor cells can proliferate before differentiating and becoming post-mitotic. Thus, delaying the time to reach the differentiation threshold increases the total number of differentiated cells. (B) Schematic showing how variable numbers of cell divisions before reaching the differentiation threshold can produce significantly different number of terminally differentiated cells while maintaining similar numbers of progenitor cells. Both scenarios maintain a pool of three progenitor cells (green cells) while producing very different numbers of differentiated cells (three versus nine). (C) Combinations of fetal bovine serum (FBS) and DMI stimuli were applied to OP9 preadipocyte cells, and differentiation was measured 96 h later as in Figure 1. The black line was drawn in manually and marks a contour line connecting stimulus conditions which maintain similar numbers of progenitor cells. As predicted in the schematics in (A) and (B), manipulating both mitogen and differentiation stimuli synegistically allows for very different numbers of terminally differentiated cells to be produced while keeping similar numbers of progenitor cells in reserve. The red dot marks conditions which produced approximately three times more adipocytes than the blue dot conditions, while still maintaining similar numbers of progenitor cells that remain undifferentiated Data shown are representative of two independent experiments.

Our finding of a molecular competition during G1 phase provides a mechanism for how the number of differentiated cells may be regulated in a tissue while preserving a similar number of progenitor cells. Maintaining an adequate of progenitor cells is important to maintain tissue health and also in the context when the same progenitor pool is used to make multiple tissue domains (Ghaben and Scherer, 2019; Sagner and Briscoe, 2019). A progenitor cell induced to differentiate first proliferates, but if it reaches the threshold to terminally differentiate, it immediately closes the proliferative window and permanently exits the cell cycle (Figures 7A and 7B). However, if the commitment to terminally differentiate is delayed, the cell can continue to divide (i.e., the proliferative window is extended). These extra divisions before differentiation commitment can lead to more differentiated cells generated per progenitor cell (Figures 7A and 7B).

We propose that a main role of the G1 competition mechanism we identified is to facilitate a balance between differentiated cells produced and progenitor cells maintained. As depicted in Figures 7A and 7B, combined mitogen and differentiation stimuli may achieve this balance by using G1 competition to regulate if and when the terminal differentiation switch is triggered and allowing control of the average number of cell cycles before differentiation commitment. Thus, by regulating the duration of G1 phase or by delaying or accelerating the rate at which PPARG levels reach the threshold to irreversibly differentiate, mitogen and differentiation stimuli may be able to control the number of differentiated cells produced while also preserving a similar number of progenitor cells that do not differentiate.

To test this model that the strengths of both mitogen and adipogenic stimuli are critical for maintaining the progenitor pool while also regulating the number of terminally differentiated cells, we performed experiments in which we systematically applied different relative strengths of adipogenic and mitogen stimuli to OP9 preadipocyte cells. We then removed the stimulus after 48 h such that we could determine at 96 h the number of cells that had terminally differentiated versus the number of cells which remained as undifferentiated progenitor cells. Markedly, increasing the concentration of mitogens resulted in both larger numbers of cells kept in the progenitor state (Figure 7C, left) and larger numbers of differentiated cells generated (Figure 7C, right), independently of the strength of the also present adipogenic stimulus . In contrast, we observed decreased numbers of progenitor cells maintained and increased numbers of differentiated cells generated when the strength of the adipogenic stimulus was increased, independently of the strength of the mitogen stimulus. In this way, there is a competition between differentiation and mitogen stimuli in G1 whereby the strength of the mitogen stimulus controls the total number of cells generated (both progenitor and differentiated), while the strength of the differentiation stimulus controls how many of the total cells remain progenitor cells or transition to the differentiated state. Taken together, these titration experiments show that in order to increase the number of differentiated cells produced while maintaining similar numbers of progenitor cells, both differentiation and mitogen stimuli must increase in tandem. This co-dependence can best be seen in the heatmaps in Figure 7C by manually plotting a contour line that connects differentiation versus mitogen stimulus conditions that maintained similar numbers of progenitor cells (Figure 7C, black curved line). The blue and red dots mark differentiation versus mitogen conditions that produce small and large numbers of terminally differentiated cells, respectively, while maintaining similar levels of progenitor cells that remain undifferentiated.

## DISCUSSION

We used adipogenesis as a model system to understand how cells coordinate terminal differentiation and cell cycle exit and developed a method to measure the precise moment of terminal differentiation while simultaneously monitoring when cells enter and exit G1 phase. With this approach, we showed that following adipogenic stimulation, progenitor cells undergo one or more cell cycles before they reach a sharp commitment point at which they terminally differentiate nearly exclusively in G1. We further show that the underlying rapid PPARG-driven switch mechanism not only commits cells to terminally differentiate but also rapidly induces expression of the CDK inhibitor p21 to force permanent exit from the cell cycle.

Mechanistically, we show that the rapid increase in p21 is mediated by PPARG-induced transcription and by expression of FKBPL and a FKBPL-mediated increase in p21 half-life. The earlier finding that p21 has a short half-life of less than 1 h at both the protein and mRNA levels (Figure 3H; Yang et al., 2017) raises a question common to all terminal differentiation processes: how can a permanent post-mitotic state be maintained? In adipogenesis, once PPARG levels reach the threshold for differentiation commitment, the upregulation of positive feedback loops cause PPARG levels to remain high independently of the input stimulus. These self-sustaining high PPARG levels permanently induce p21 and thus permanently prevent cells from re-entering the cell cycle. We also showed that knocking down p21 after cells have passed the threshold for terminal differentiation re-activates the cell cycle but also results in mitotic defects, arguing that maintaining high p21 levels after the commitment to differentiate is critical for cell health. Notably, in our experiments we plated cells at low density conditions such that p21 was the dominant CDK inhibitor. At higher cell densities, the p21 homolog p27 is expected to have a synergistic role along with p21 in regulating CDK activity and terminal cell differentiation.

Importantly, our experiments revealed that the length of G1 and number of cell divisions are regulated during the differentiation process by a competition mechanism between cyclin D1 and PPARG-induced expression of p21. This competition during G1 phase controls whether a cell enters the next cell cycle or terminally differentiates. At the same time, we found that PPARG expression is delayed by repression during the S/G2/M period of the cell cycle and that the self-amplification of PPARG becomes only gradually sensitized over a 36-h-long time period following adipogenic stimulation. As a consequence of regulating these different timing mechanisms, we show that cells delay or accelerate the time when they commit to terminally differentiate, which in turn controls the number of cell cycles per progenitor cell and the number of terminal differentiated cells produced on average from progenitor cells.

Finally, we show that this molecular competition during G1 can be regulated by the strength of both external adipogenic and mitogen stimuli and that increasing either stimuli can increase the number of terminally differentiated cells (Figures 6 and 7; see also Ahrends et al., 2014; Park et al., 2012). However, adipogenic stimuli increase the number of differentiated cells by directly depleting the existing number of progenitor cells, while mitogen stimuli increase the number of differentiated cells by first generating a large pool of progenitor cells out of which only a few cells differentiate (Figure 7). This dual control mechanism can explain the conundrum of how mice lacking p21, which mimics a state of higher mitogen signaling, can have significantly larger numbers of adipocytes than control mice, despite proliferation and terminal differentiation being opposing processes (Naaz et al., 2004). Furthermore, we demonstrate that mitogen and adipogenic stimuli can coordinate a robust terminal differentiation process while ensuring that a pool of progenitor cells is maintained. Intriguingly, identifying such a mechanism to regulate the balance of progenitor and terminally differentiated cells is particularly relevant in the context of generating complex organs such as the spinal cord, in which the different neuronal domains along the dorsal-ventral axis are formed through terminal differentiation of different cell types in a temporal sequence from the same progenitor pool (Molina and Pituello, 2017; Sagner and Briscoe, 2019). Not having enough terminally differentiated cells or an abnormally depleted progenitor pool at any stage in development could lead to deformities and disease.

Together, the opposing roles of cyclin D1 and p21 in regulating the time when cells commit to terminally differentiate (Figure 6), and the large *in vivo* effect of p21 and p27 knockouts on fat mass (Naaz et al., 2004), suggest that therapeutic strategies aimed at regulating the time when cells commit to terminally differentiate may prove useful to control the size of terminally differentiated tissues. For example, as DNA damage and aging increase p21 expression, such conditions may cause an imbalance between progenitor and differentiated cells and may result in insufficient fat and other terminally differentiated cells produced through this competition mechanism. It is suggestive to propose that our finding may represent an example of a more general control principle whereby the size of tissues may generally be regulated by a competition between cyclins on one side and drivers of terminal cell differentiation and CDK inhibitors on the other side.

## STAR★METHODS

### RESOURCE AVAILABILITY

#### Lead Contact

Further information and requests for resources and reagents should be directed to and will be fulfilled by the Lead Contact, Dr. Mary N. Teruel (mnt4002@med.cornell.edu).

#### Materials Availability

Further information and requests for reagents may be directed to, and will be fulfilled by Dr. Mary N. Teruel.

#### Data and Code Availability

MATLAB analysis scripts and datasets used in this paper are available on Medeley Data at the following: https://doi.org/10.17632/n9r24r9tdb.1

### EXPERIMENTAL MODEL AND SUBJECT DETAILS

The OP9 mouse stromal cell line (ATCC CRL-2749) used in this manuscript was established from newborn op/op mouse calvaria and are classified as being from bone marrow/stroma tissue and of an embryonic stem cell/macrophage cell type. The OP9 cells were maintained according to protocols described in Bahrami-Nejad et al. (2018) and Wolins et al. (2006).

### METHOD DETAILS

#### Generation of PPARG and APC/C dual-reporter cell line

OP9 cells with endogenously tagged citrine-PPARG2 and stably infected H2B-mTurqoise were generated as previously described (Bahrami-Nejad*, Zhao*, et al., 2018). To make dual reporter cells, a lentiviral strategy, outlined below, was used to stably introduce the APC/C reporter into the citrine-PPARG2 cell line.

Lentiviral production was carried out using a third-generation lentiviral packaging system that consisted of the following packaging vectors: pMDlg, pCMV-VSVG, and pRSV-Rev and the lentiviral vector with the APC/C reporter, pCSII-EF-Geminin(1-110)-mCherry. The vectors were transfected into (12 μg pMDlg, 6 μg pRSV-Rev, 3 μg pCMV-VSVG, 6 μg lentiviral vector of interest) HEK293T growing in Opti-MEM with Lipofectamine 2000 using the manufacturer’s suggested protocol. HEK293T cells were seeded one day before transfection at a density of 5x10^6^ cells in a collagen treated 10 cm cell culture dish. The transfection media was removed after one day and replaced with 7 mL of fresh Opti-MEM. Viral particles were collected over two days by collecting the supernatant and replacing it with fresh Opti-MEM every day. The final supernatant (total volume ~14 mL) was then filtered through a 0.45 μm filter to remove cellular debris and then concentrated using Amicon Ultra-15 Centrifugal Filter tubes with a 100,000 kDA molecular weight cutoff (EMD Millipore) spun at 3,300 r*cf.* for 30 minutes. Concentrated viral supernatant was then aliquoted into four or five cryogenic freezing tubes containing 40 μL each for storage at −80°C.

To infect cells with lentivirus, OP9 cells were first seeded at a density of 50,000 cells per well of a 6 cm cell culture plate. One aliquot of concentrated viral supernatant was mixed in 2 mL of MEM-α media (ThermoFisher Scientific) containing 100 units/mL Penicillin, 100mg/mL Streptomycin, and 292 mg/mL L-glutamate supplemented with 10% FBS. Polybrene was added at a concentration of 7 μg/mL to facilitate infection efficiency. After one day, infection medium was changed to fresh growth medium (MEM-α media (ThermoFisher Scientific) containing 100 units/mL Penicillin, 100mg/mL Streptomycin, and 292 mg/mL L-glutamate supplemented with 20% FBS). Upon reaching confluence, infected cells were trypsin detached and seeded in larger T75 flasks for expansion prior to sorting. After two days of growth, cells were trypsin detached and submitted for FACS sorting to obtain a population of APC/C sensor positive cells marked by positive mCherry fluorescence.

#### Generation of a PPARG, APC/C activity reporter, and CDK2 triple reporter cell line

Lentivirus was generated for the CDK2 sensor from the vector pCSII-EF-DHB-mTurquoise (gift from the lab of Tobias Meyer) in the same manner described above and used to infect cells expressing citrine-PPARG, APC/C activity reporter (mcherry), and H2B-iRFP670 (far red) cells. Selection of triple-reporter cells was done by FACS sorting for cells that were positive for both mCherry and mTurquoise.

#### Generation of a PPARG and CRL4-CDT dual reporter cell line

The CRL4-Cdt2 construct was developed by Atsushi Miyawaki’s lab (Sakaue-Sawano et al., 2017) and was obtained from the lab of Tobias Meyer. We changed the fluorescent tag to iRFP670 and generated lentivirus in the same manner described above. Selection of triple-reporter cells stably expressing iRFP670-CRL4-Cdt2 was done by FACS sorting for cells that were positive for both mCherry and iRFP670.

#### Cell culture and differentiation

Wild-type and reporter OP9 cell lines were cultured according to previously published protocols (Ahrends et al., 2014; Bahrami-Nejad et al., 2018; Wolins et al., 2006). Briefly, the cells were cultured in growth media consisting of MEM-α media (ThermoFisher Scientific) containing 100 units/mL Penicillin, 100mg/mL Streptomycin, and 292 mg/mL L-glutamate supplemented with 20% FBS. To induce differentiation, two methods were used. The first method is the standard 96-hour DMI protocol commonly used to induce adipogenesis (Bahrami-Nejad et al., 2018; Wu et al., 1999). An adipogenic cocktail (DMI) consisting of dexamethasone (1 μM, Sigma-Aldrich), IBMX (125 μM, Sigma-Aldrich), and insulin (1.75 nM, Sigma-Aldrich) is added to the cell media for 48 hours, and then aspirated and replaced with fresh media containing 1.75 nM insulin for another 48 hours. In the second method, 1 μM of Rosiglitazone (Cayman, USA) is added to the cell media for 48 hours, and then aspirated and replaced with fresh media containing 1.75 nM insulin for another 48 hours. For fixed cell experiments, the media consisted of the growth media described above with one modification: 10% FBS was used (instead of 20% FBS) during differentiation conditions. The one exception is the reduced serum experiments in Figure 4D, in which 2% FBS was used in the growth media during differentiation. For all live cell experiments, the differentiation stimuli were added to Fluorobrite DMEM media (ThermoFisher Scientific) containing 100 units/mL Penicillin, 100mg/mL Streptomycin, and 292 mg/mL L-glutamate supplemented with 10% FBS. The MEK inhibitor PD0325091 was used at a final concentration of 100 nM.

In our experiments, we purposely used sub-confluent cell plating conditions in order to maximize the number of cell divisions, to reduce the effect of cell density on cell cycle arrest, and to improve the fidelity of the automated tracking algorithm (Figures S3A and S3B). Because we plated cells at low density conditions, p21 was the dominant CDK inhibitor. At higher cell densities, the p21 homolog p27 is expected to have a synergistic role along with p21 in regulating CDK activity and terminal cell differentiation. Such a synergistic role of both CDK inhibitors is consistent with knockout data in mice which showed a 6-fold increase in fat mass when p27 and p21 were knocked out together as compared to smaller increases with only individual knockouts (Naaz et al., 2004).

#### siRNA-mediated gene silencing

siRNA targeting *Pparg, Cebpa, p21, CyclinD1, Fkbpl* and the AllStars Negative Control siRNA were purchased from QIAGEN. For siRNA knockdown in the live-cell imaging experiments in dual-reporter cells (Figure 4, 5A, 5D, and 5E), OP9 cells were transfected by reverse-transfection using μL Lipofectamine RNAiMax (Invitrogen). Briefly, our reverse-transfection protocol per well is as follows: mixed 20 μL of Optimem, 0.5 μL of a 10 μM siRNA stock solution, and 0.3 μL of RNAiMax. Let solution incubate at room temperature for 10 minutes and then add 80 μL of culture media containing the desired number of cells per well. Then the entire (~100μL) volume is plated into one well of a 96-well plate. The siRNA/RNAiMax mixture was left on the cells for 24 hours before being aspirated away and replaced with fresh culture media containing DMI to begin the differentiation protocol.

For the live-cell imaging experiments in dual-reporter cells transfected at the 48-hour time point (Figure 6), the following protocol per well was used: siRNA mixture was prepared using 0.6 μL Lipofectamine RNAiMAX, 0.5 μL of a 10 μM siRNA stock solution, and 20 μL of Optimem. Incubate the mixture for 10 minutes then add 180 μL of Fluorobrite media consisting of 1.75 nM insulin. The entire solution (~200μL total volume) was then added to cells at the 48-hour time point and left on until the end of the experiment.

#### Overexpression of p21

A retroviral vector containing DHFR-Chy-p21(Spencer et al., 2013) (gift from the lab of Tobias Meyer) was used to generate viral particles to stably infect DHFR-Chy-p21 into a modified dual-reporter cell line. This cell line was also stably infected with H2B-iRFP670 and a version of the APC/C reporter fused to mCerulean3. Positive clones were selected for by FACS in cell culture media containing 10 μM TMP. Cells were sorted into culture media with no TMP and grown in the absence of TMP. All overexpression experiments were done by adding 10 μM TMP into the culture media or differentiation media. In control experiments, 10 μM DMSO was added instead of TMP.

#### Immunofluorescence (IF) staining

All cultured cells were fixed with 4% PFA in PBS for 30 min at room temperature, followed by five washes with PBS using an automated plate washer (Biotek). Cells were then permeabilized with 0.1% Triton X-100 in PBS for 15 minutes at 4°C, followed by blocking for 1 hour in 5% bovine serum albumin (BSA, Sigma Aldrich) in PBS. The cells were incubated with primary antibodies in 2% BSA in PBS overnight at 4°C: mouse anti-PPARγ (Santa Cruz Biotech, sc-7273, 1:1,000), rabbit anti-CEBPα (Santa Cruz Biotech, sc-61, 1:1,000), mouse anti-p21 (Santa Cruz Biotech, sc-6246, 1:100), cyclinD1 (Abcam, ab137145, 1:1,000), adiponectin (Abcam, ab22554, 1:1,000), Glut4 (Santa Cruz Biotech, sc-1608, 1:500), FABP4 (R&D Systems, AF1443, 1:1,000). After washing, cells were incubated with Hoechst (1:20,000) and secondary antibodies in 2% BSA / PBS for 1 hour. Secondary antibodies included Alexa-Fluor-conjugated anti-rabbit, anti-mouse, and anti-goat antibodies (Thermo Fisher Scientific). All secondary antibodies were used at a 1:1,000 dilution. Where indicated, neutral lipids were measured by adding HCS LipidTOX Deep Red Neutral Lipid Stain 637/655 (1:1,000), ThermoFisher Scientific H34477) to secondary antibody solution. Cells were washed five times with PBS in an automated plate washer prior to imaging. For fixed-cell time course experiments, approximately 7,000 wild-type or dual-reporter OP9 cells were used to calculate mean values at each time point for each technical replicate.

#### RNaseq

siRNA targeting Pparg (# L-040712-00-0005) and Negative Control siRNA(# D-001810-10-05) were purchased from Dharmacon and transfected into OP9 cells using Lipofectamine RNAiMax (Invitrogen) according to the manufacturer’s protocol. siRNA was used at a concentration of 25 nM, and the RNAiMAX/siRNA mixture was applied for 48 hours prior to the induction of differentiation. For gene expression analysis of OP9 cell samples, the cells were differentiated for 144 hours using a previously described protocol (Ahrends et al., 2014). RNA from three independent biological experiments were collected at different time points before and after induction of differentiation including (d0-d6) the extraction was completed using RNeasy Mini Kit (QIAGEN, Cat. 74104). RNA quality of all samples (n = 7 time points and n = 3 experiments from independent passages) was evaluated by both Nanodrop for (A260/280 > 2) and Bioanalyzer 2100 High Sensitivity RNA Analysis chips (Agilent, Cat. 5067-1513) which displayed intact RNA integrity (RIN > 9). mRNA samples were concentrated to ≤ 5 μl by MinElute column (QIAGEN, Cat. 74204). For generation of RNA-seq libraries, polyadenylated mRNA was isolated from 300 ng of total RNA by incubation with oligo-DT attached magnetic beads and followed by strand-specific library preparation using the TruSeq Stranded mRNA Library Preparation kit (Ilumina, Cat. 20020595). Briefly, isolated polyadenylated mRNA was fragmented using divalent cations under elevated temperature and 1^st^ and 2^nd^ strands DNA were synthesized using Superscript II Reverse Transcriptase (provided with Ilumina kit). A-tailing and adaptor ligation was performed according to the manufacturer’s protocol; the resulting dsDNA was enriched in a PCR reaction based on predetermined CT values and cleaned using AMPure XP beads (provided with Ilumina kit). Concentrations of enriched dsDNA fragments with specific adapters were determined and base pair average size as well as library integrity were analyzed using the Bioanalyzer DNA High Sensitivity chips (Agilent, Cat. 5067-4626). Samples were pooled and sequenced on the Illumina NextSeq 500/550 High Output platform (Illumina, FC-404-2002) up to 18 samples per lane with 1% PhiX spike as a control.

The read quality of the raw FASTQ files was checked with FastQC (Andrews, 2010) (v0.11.7). Next, reads were pseudo-aligned to the mouse reference transcriptome (Mus_musculus.GRCm38.cdna) using Kallisto(Bray et al., 2016) (v0.44.0) with the quantification algorithm enabled, the number of bootstraps set to 100, and run in paired-end mode. The Kallisto output files were read into R using Sleuth, and the transcripts per million (TPM), a measurement of the proportion of transcripts in the RNA pool, was used for downstream differential expression analysis(Pimentel et al., 2017).

#### Measuring protein decay rates using cycloheximide

Protein decay rates were quantified as previously described(Bahrami-Nejad et al., 2018). Briefly 10,000 OP9 cells were seeded in 96-well plates) one plate for each time point. Cells were induced to differentiate with DMI for 24 hours. Cyclohexamide was added to the media at a final concentration of 30 μM. Cells were fixed and stained at different times after addition of cyclohexamide, and immunofluorescence was used to quantify protein concentration. Half-lives were obtained by fitting first order exponential decay curves to the data.

#### Fluorescent imaging

Imaging was conducted using an ImageXpress MicroXL (Molecular Devices, USA) with a 10X Plan Apo 0.45 NA objective. Live fluorescent imaging was conducted at 37°C with 5% CO_2_. A camera bin of 2x2 was used for all imaging condition. Cells were plated in optically clear 96-well plates: plastic-bottom Costar plates (#3904) for fixed imaging or Ibidi μ-Plate (#89626) for live imaging. Living cells were imaged in FluoroBrite DMEM media (Invitrogen) with 10% FBS, 1% Penicillin/Streptomycin and insulin to reduce background fluorescence. Images were taken every 12 min in different fluorescent channels: CFP, YFP and/or RFP. Total light exposure time was kept less than 700 ms for each time point. Four, non-overlapping sites in each well were imaged. Cell culture media were changed at least every 48h.

### QUANTIFICATION AND STATISTICAL ANALYSIS

#### Imaging data processing

Data processing of fluorescent images was conducted in MATLAB R2016a (MathWorks). Unless stated otherwise, fluorescent imaging data were obtained by automated image segmentation, tracking and measurement using the MACKtrack package for MATLAB. Quantification of PPARG- and CEBPA-positive cells in fixed samples was based on quantification of mean fluorescence signal over nuclei. Cells were scored as PPARG- and CEBPA-positive if the marker expression level was above a preset cut-off determined by the bimodal expression at the end of the experiment.

For live imaging data of OP9 cells, the CFP channel capturing H2B-mTurqoise fluorescence was used for nuclear segmentation and cell tracking. Obtained single-cell traces were filtered to removed incomplete or mistracked traces according to the following criteria: cells absent within 6 hours of the endpoint, cell traces that started more than 4 hours after the first time point, cells that had large increase or decrease in PPARG intensity normalized to the previous time point, cells where H2B drops did not match drops in the APC/C reporter. If cells were binned according to their PPARG expression, cells were binned based on their mean nuclear PPARG expression at the described time points.

The percent of cells in the S/G2/M phases at each time point is calculated by counting the cells that expressed the APC/C reporter during the 96-hour differentiation period divided by the total number of cells. The percent of PPARG high cells was assessed by counting cells that above the PPARG threshold at that time point and dividing by the total number of cells at that time point.

#### Estimating a differentiation commitment point (i.e., PPARG threshold)

PPARG values at the end of a differentiation experiment typically exhibit a bimodal distribution. In order to estimate a commitment point, PPARG values at the last frame of the experiment was fit to a 2 component Gaussian mixture model. Cells were then classified as either differentiated or undifferentiated based on whether they more closely associated with the high or low component of the mixture model, respectively. The commitment point was then assessed as the value of PPARG at the 48-hour time point, before the stimuli was removed, that predicted the final differentiation classification with a false positive rate of less that 5%. In experiments where multiple conditions are present, the Gaussian mixture model was only fitted to the negative control and the commitment point was selected based on the negative control model and applied to all other conditions in the same experiment.

Note that the early in adipogenesis before cells reach the threshold, PPARG levels are not correlated with endpoint measurements of adipocyte markers (Figure 1G). However, once the threshold was reached, PPARG levels sharply switch to being positively correlated, supporting that crossing the PPARG threshold marks a short time window of PPARG self-amplification that causes an irreversible commitment to the future terminally differentiated adipocyte state (see also Figure S1D). Notably, without aligning time courses to a threshold, we would only see that there is an increased probabilistic relationship between PPARG expression and mature adipocyte markers; for example, see rightmost plot in Figure 1G which shows a gradual increase in correlation with PPARG with GLUT4 when time courses are not aligned by the threshold. Thus, without being able to measure a threshold for each cell and being able to align the PPARG time course for each cell by this threshold, we would be unable to mark a precise time point for differentiation commitment, as can be seen when comparing the aligned and unaligned plots in Figures 1G and S1E.

#### Statistics

Unless specified otherwise, data are expressed as mean ± standard error of the mean (SEM). Live traces are expressed as median ± interquartile range (25^th^-75^th^ percentiles). For histograms with a y axis labeled “Fraction of Cells,” each histogram (not each plot) is normalized to the total number of cells in the population of that histogram such that all bars in the histogram add to 1. Representative results are representative of at least two independent experiments.

#### Data availability

All relevant data from this manuscript are available upon request.

## Supplementary Material

1

2

3

## Figures and Tables

**Table:** KEY RESOURCES TABLE

REAGENT or RESOURCE	SOURCE	IDENTIFIER
Antibodies		
Mouse monoclonal anti-PPARgamma (E-8)	Santa Cruz Biotechnology	Cat# sc-7273
Rabbit polyclonal anti-PPARG (81B8)	Cell Signaling	Cat# 2443
Mouse monoclonal anti-p21 (F5)	Santa Cruz Biotechnology	Cat# sc-6246
Rabbit polyclonal anti-C/EBPα	Santa Cruz Biotechnology	Cat# sc61
Goat polyclonal anti-FABP4	R & D Systems	Cat# AF1443
Mouse monoclonal anti-Adiponectin	Abcam	Cat# ab22554
Goat polyclonal anti-Glut4	Santa Cruz Biotechnology	Cat# sc-1608
Goat anti- Rabbit IgG (H+L) cross-adsorbed secondary antibody, Alexa Fluor 514	Invitrogen	Cat# A31558
Goat anti- Rabbit IgG (H+L) cross-adsorbed secondary antibody, Alexa Fluor 647	Invitrogen	Cat# A21244
Goat anti- Mouse IgG (H+L) cross-adsorbed secondary antibody, Alexa Fluor 594	Invitrogen	Cat# A11032
Goat anti- Mouse IgG (H+L) cross-adsorbed secondary antibody, Alexa Fluor 647	Invitrogen	Cat# A21235
Donkey anti- Mouse IgG (H+L) cross-adsorbed secondary antibody, Alexa Fluor 647	Invitrogen	Cat# A31571
Chemicals, Peptides, and Recombinant Proteins		
IBMX	Sigma-Aldrich	Cat# 7018
Dexamethasone	Sigma-Aldrich	Cat# D1756
Insulin	Sigma-Aldrich	Cat# I6634
Saponin	Sigma-Aldrich	Cat# 47036
Bovine Serum Albumin	Sigma-Aldrich	Cat# 7906
Rosiglitazone	Cayman Chemical Company	Cat# 7906
Trimethoprim (TMP)	Cayman Chemical Company	Cat# 16473
HCS LipidTOX Deep Red neutral lipid stain	Thermo Fisher Scientific	Cat# H34477
Experimental Models: Cell Lines		
OP9 mouse stromal	Wolins et al., 2006	N/A
citrine(YFP)-PPARG OP9 cells	Bahrami*,Zhao*, et al., 2018	N/A
Dual reporter citrine-PPARG and APC/C-reporter-mcherry (RFP) OP9 cells	This paper	N/A
Triple reporter citrine-PPARG, APC/C-reporter-mcherry (RFP), CDK2 activity sensor (CFP) OP9 cells	This paper	N/A
Dual reporter citrine-PPARG and CRL4-Cdt2-mcherry (RFP) OP9 cells	This paper	N/A
siRNA Sequences		
See Table S1		N/A
Recombinant DNA		
Plasmid: CSII-EF1-H2B-mTurquoise	Gift from Tobias Meyer Laboratory	N/A
Plasmid: CSII-EF1-H2B-iRFP670	This paper	N/A
Plasmid: CSII-EF-MCS-mCherry-Geminin1to110	Gift from Tobias Meyer Laboratory	N/A
Plasmid: CSII-EF-MCS-mCerulean-Geminin1to110	Gift from Tobias Meyer Laboratory	N/A
Plasmid: pLV-EF1a-hDHB-mTurquoise-Blast	Gift from Tobias Meyer Laboratory	N/A
Plasmid: pCru5-6.10-TCT-ACC-DHFR-p21-IRES-puro	Gift from Tobias Meyer Laboratory	N/A
Plasmid: pCru5-6.10-TCT-ACC-DHFR-mChy-IRES-puro	Gift from Tobias Meyer Laboratory	N/A
Plasmid: iRFP670-CRL4-Cdt2	This paper	N/A

## Highlights

- Cells simultaneously terminally differentiate and permanently exit the cell cycle in G1
- Differentiation occurs by a competition in G1 that lasts over multiple cell cycles
- The G1 competition also controls the number of progenitor cells maintained
